# Dynamin-2 reduction rescues the skeletal myopathy of a SPEG-deficient mouse model

**DOI:** 10.1172/jci.insight.157336

**Published:** 2022-08-08

**Authors:** Qifei Li, Jasmine Lin, Jeffrey J. Widrick, Shiyu Luo, Gu Li, Yuanfan Zhang, Jocelyn Laporte, Mark A. Perrella, Xiaoli Liu, Pankaj B. Agrawal

**Affiliations:** 1 Division of Newborn Medicine,; 2 Division of Genetics and Genomics, and; 3 The Manton Center for Orphan Disease Research, Boston Children’s Hospital, Harvard Medical School, Boston, Massachusetts, USA.; 4 Division of Pulmonary and Critical Care Medicine and; 5 Department of Pediatric Newborn Medicine, Brigham and Women’s Hospital, Harvard Medical School, Boston, Massachusetts, USA.; 6 Institute of Genetics and Molecular and Cellular Biology (IGBMC), INSERM U1258, CNRS UMR7104, Strasbourg University, Illkirch, France.

**Keywords:** Muscle Biology, Therapeutics, Gene therapy, Mouse models, Muscle

## Abstract

Striated preferentially expressed protein kinase (*SPEG*), a myosin light chain kinase, is mutated in centronuclear myopathy (CNM) and/or dilated cardiomyopathy. No precise therapies are available for this disorder, and gene replacement therapy is not a feasible option due to the large size of *SPEG*. We evaluated the potential of dynamin-2 (DNM2) reduction as a potential therapeutic strategy because it has been shown to revert muscle phenotypes in mouse models of CNM caused by *MTM1*, *DNM2*, and *BIN1* mutations. We determined that SPEG-β interacted with DNM2, and SPEG deficiency caused an increase in DNM2 levels. The DNM2 reduction strategy in *Speg*-KO mice was associated with an increase in life span, body weight, and motor performance. Additionally, it normalized the distribution of triadic proteins, triad ultrastructure, and triad number and restored phosphatidylinositol-3-phosphate levels in SPEG-deficient skeletal muscles. Although DNM2 reduction rescued the myopathy phenotype, it did not improve cardiac dysfunction, indicating a differential tissue-specific function. Combining DNM2 reduction with other strategies may be needed to target both the cardiac and skeletal defects associated with SPEG deficiency. DNM2 reduction should be explored as a therapeutic strategy against other genetic myopathies (and dystrophies) associated with a high level of DNM2.

## Graphical abstract

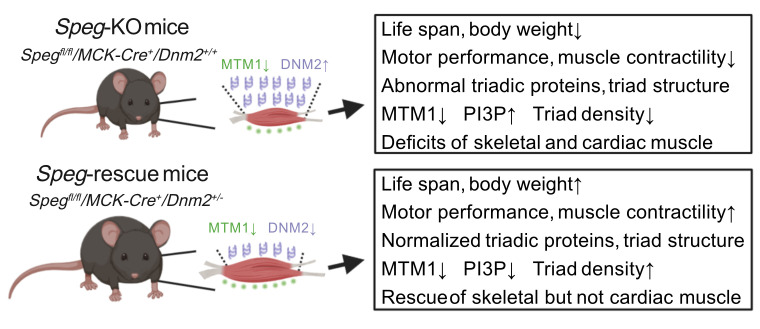

## Introduction

Centronuclear myopathy (CNM), a type of congenital myopathy (CM), is clinically characterized by hypotonia and muscle dysfunction ranging in severity from mild delays in motor milestones to fatal weakness of respiratory organs (1). The histopathological and molecular findings of CNM include increase in central nucleation of myofibers, variability of myofiber size, disruptions in the triad structure, and defective excitation-contraction coupling (1–3). Mutations in genes including *MTM1* (MIM 300415; myotubularin), *DNM2* (MIM 602378; dynamin 2), *BIN1* (MIM 601248; bridging integrator 1), *RYR1* (MIM 180901; ryanodine receptor 1), *CACNA1S* (MIM 114208; alpha 1s subunit of the dihydropyridine receptor, DHPR), *TTN* (MIM 188840; titin), and *SPEG* (MIM 615950; striated preferentially expressed protein kinase) have been identified for over 60% of patients with CNM (4, 5).

We have previously demonstrated that constitutive SPEG-deficient mice develop dilated cardiomyopathy (DCM), and a majority of them die in utero or shortly after birth due to heart failure (6). To overcome the perinatal lethality, we created floxed *Speg* mice and crossed them with muscle creatine kinase (MCK) Cre-expressing mice, which give rise to striated muscle-specific *Speg*-KO mice wherein Cre expression starts at embryonic day 17, peaks at postnatal day 10, and remains high thereafter (7). *Speg*-KO mice demonstrate poor skeletal and cardiac function along with defective triad formation, abnormal excitation-contraction coupling, and calcium mishandling in skeletal muscles also seen in other CNM (4, 7).

CMs and cardiomyopathies can coexist in certain genetic conditions (8, 9), which complicates therapeutic strategies, especially if the protein is large with diverse genetic mutations and variable tissue-specific functional consequences. *SPEG* is one such gene, which encodes 2 large proteins (260 kDa and 350 kDa for SPEG-α and SPEG-β, respectively), and when mutated, causes CNM and/or DCM in humans (3, 5, 10, 11). SPEG belongs to the myosin light chain kinase protein family, which are involved in the structure and regulation of cytoskeletal function in myocytes (12). SPEG-α and SPEG-β contain a variable number of Ig-like, 3 fibronectin type III, and 2 tandemly arranged serine/threonine kinase domains arranged in tandem and are predominantly expressed in skeletal and cardiac muscles (13). Patients with recessive mutations affecting both SPEG-α and SPEG-β isoforms demonstrated both CNM and DCM, with the disease being less severe if one or both variants affect only SPEG-β, the larger isoform (3, 5, 11). To date, over 21 patients have been identified with recessive *SPEG* mutations, with 11 deaths associated with respiratory or cardiac failure. Unfortunately, no precise treatments are available for such disorders, and there is an urgent need to develop them.

One of the other genes mutated in CNM is *DNM2* that encodes dynamin-2 (DNM2), a large ubiquitously expressed GTPase protein implicated in membrane remodeling, endocytosis, and cytoskeleton organization (14, 15). Elevated levels or activity of DNM2 have been reported as a consequence of mutations in *MTM1*, *DNM2*, and *BIN1*, all associated with CNM (16–19). Reducing DNM2 levels can rescue the CNM phenotypes of *Mtm1*-KO, *Bin1*-KO, and *Dnm2*-knockin mice (16, 18, 20–24), suggesting that these CNM proteins may participate in an interdependent functional network (4, 25, 26).

The goal of our study was to test if DNM2 reduction can be an effective strategy to rescue SPEG deficiency. The rationale included: 1) *MTM1*, *DNM2*, *BIN1*, and *SPEG* mutations cause CNM (5, 19, 27, 28); 2) mouse models of CNM genes show an abnormal triad structure and defective calcium handling in skeletal muscles (7, 19, 29, 30); and 3) in this study we show that DNM2 levels were increased in SPEG-deficient mice and SPEG-β interacted with DNM2. We elucidated that reducing DNM2 levels in SPEG-deficient mice could rescue the skeletal muscle phenotype.

## Results

### SPEG-β interacts with DNM2 and its deficiency causes an increase in DNM2 levels.

**Figure 1:**
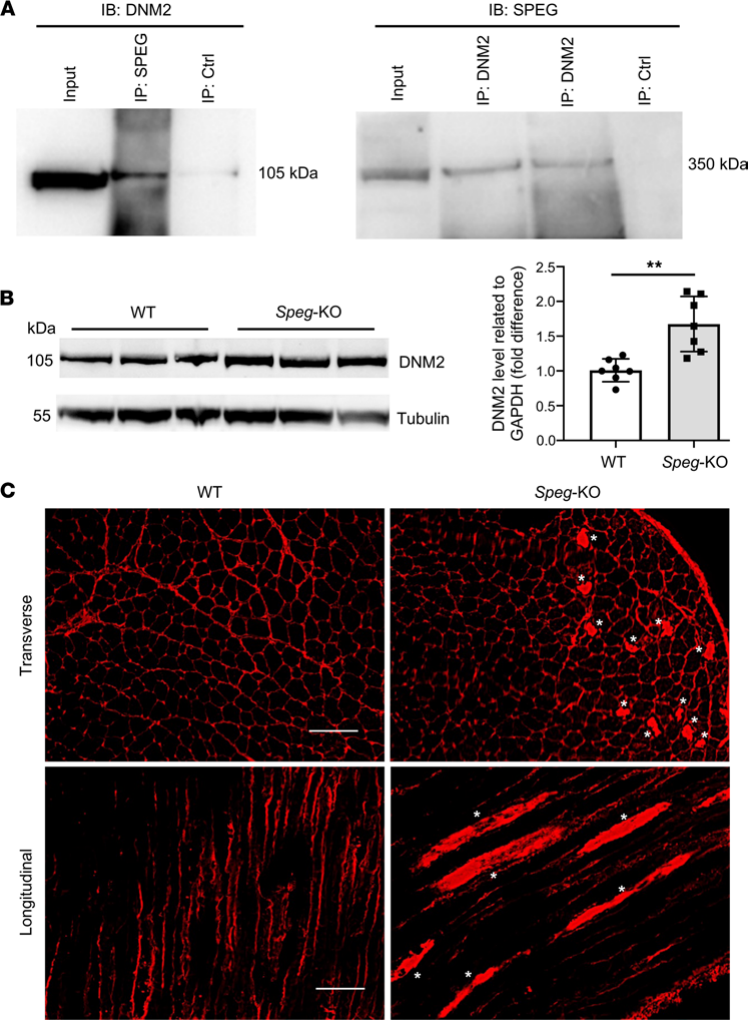
DNM2 expression and distribution in skeletal muscle from *Speg*-KO and WT mice. (**A**) SPEG-β and DNM2 coimmunoprecipitated from differentiated C2C12 myotube lysates with the use of rabbit anti-SPEG generated against a FLAG-tagged aortic preferentially expressed gene-1 fusion protein and anti-DNM2 antibodies. (**B**) Western blot analysis for DNM2 protein in *Speg*-KO skeletal muscles. Left panel shows representative image of DNM2 protein in *Speg*-KO versus WT quadriceps. Tubulin is used as a loading control. Right panel represents quantification of DNM2 expression relative to the expression of tubulin. *Speg*-KO mice demonstrated an average of 1.7-fold increase in DNM2 expression over WT in quadriceps and gastrocnemius muscles (**P ˂ 0.01, *n* = 7 per group; unpaired 2-tailed *t* test). (**C**) Immunostaining for DNM2 protein in tibialis anterior (TA) muscle from *Speg*-KO and WT mice (over 100 fibers were analyzed from each group, *n* = 3 per group). *Speg*-KO mice displayed an abnormal DNM2 accumulation (denoted by asterisks). Scale bars: 100 μm.

To test for potential interaction between SPEG and DNM2, we performed coimmunoprecipitation (co-IP) experiments using differentiated C2C12 myotube, soleus, and triceps lysates. Full-length SPEG (including both SPEG-β and SPEG-α) and DNM2 co-immunoprecipitated with each other using anti-SPEG and anti-DNM2 antibodies, confirming their interaction between DNM2 and SPEG-β exclusively (Figure 1A and Supplemental Figure 1; supplemental material available online with this article; https://doi.org/10.1172/jci.insight.157336DS1).

To evaluate the effects of SPEG deficiency on DNM2 levels, skeletal muscles from wild-type (WT) and *Speg*-KO mice were tested. We found that *Speg*-KO mice had a 1.7-fold increase in DNM2 level over WT (Figure 1B). Additionally, transverse and longitudinal sections of tibialis anterior (TA) muscle in *Speg*-KO mice displayed an abnormal accumulation of DNM2 (Figure 1C). Interestingly, elevated levels or activity of DNM2 have been reported as a consequence of mutations in *MTM1*, *BIN1*, and *DNM2* causing CNM (16–18).

### Generating Speg-KO/DNM2^+/–^ mice to test rescue potential of DNM2 reduction strategy.

**Figure 2:**
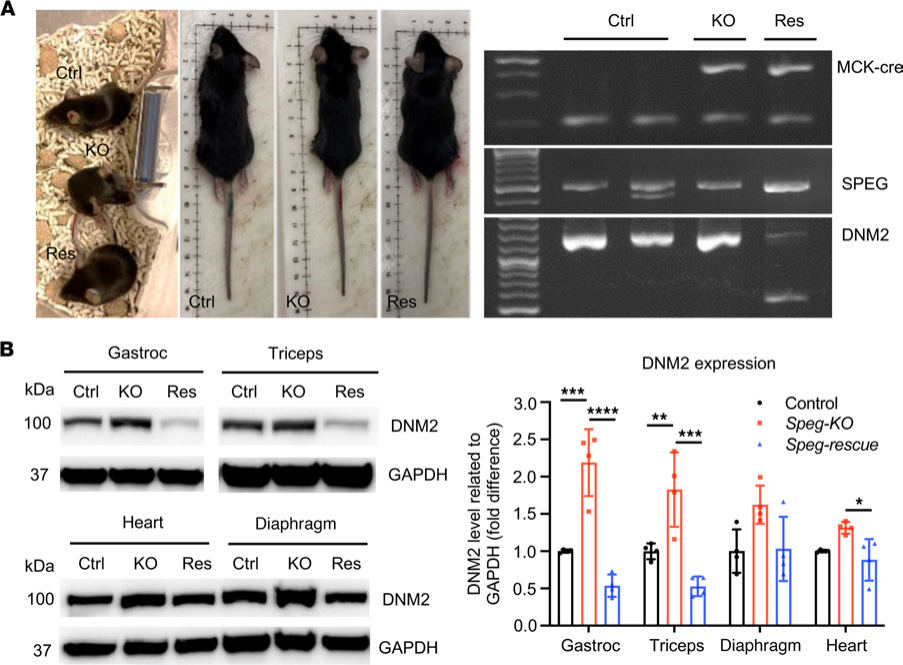
Generation of *Speg*-KO mice with DNM2 haploinsufficiency. (**A**) Representative images of control, *Speg*-KO, and *Speg*-rescue mice at 3 months of age and agarose gel analysis of DNA isolated from tails of mice showing the presence or absence of *MCK-Cre* (top), floxed *Speg* (middle), and *DNM2* (bottom). *MCK-Cre*^+^ mice displayed bands for the transgene (~450 bp) and internal positive control (200 bp) while the floxed *Speg* allele is 485 bp in size versus 422 bp for the WT. *DNM2*-heterozygous mice displayed 2 bands at 553 bp and 1432 bp versus 1 band at 1432 bp for the WT. Ctrl, control; KO, *Speg*-KO; Res, *Speg*-rescue. (**B**) Immunoblot analysis and quantification of DNM2 in various types of striated muscles, including gastrocnemius (gastroc), triceps, diaphragm, and heart. (*P ˂ 0.05; **P ˂ 0.01, ***P ˂ 0.001, ****P < 0.0001, *n* = 4 per genotype; 1-way ANOVA with Tukey’s post hoc test.)

To evaluate if DNM2 reduction may rescue *Speg*-KO mice, we first generated *Speg*-KO mice that were DNM2 haploinsufficient (*Speg^fl/fl^ MCK-Cre^+^ Dnm2^+/–^*; *Speg*-rescue). The breeding strategy is shown in Supplemental Figure 2, and breeding outcome for *Speg*-rescue mice is listed in Supplemental Table 1. Representative images of control, *Speg*-KO, and *Speg*-rescue mice at 3 months and genotyping strategy are shown in Figure 2A. To further evaluate the effects of *Dnm2* haploinsufficiency at the protein level, different types of striated muscles, including the diaphragm, heart, gastrocnemius (gastroc), and triceps muscles, were collected at 12 weeks of age from *Speg*-rescue, *Speg*-KO, and control mice. The amount of DNM2 protein was lower in the *Speg*-rescue versus the *Speg*-KO mice, especially in gastroc and triceps muscles (showing 76% and 71% decrease, respectively) compared with diaphragm and heart (showing 36.5% and 33% reduction, respectively) (Figure 2B and Supplemental Figure 3).

### DNM2 reduction improves survival and increases body weight.

**Figure 3:**
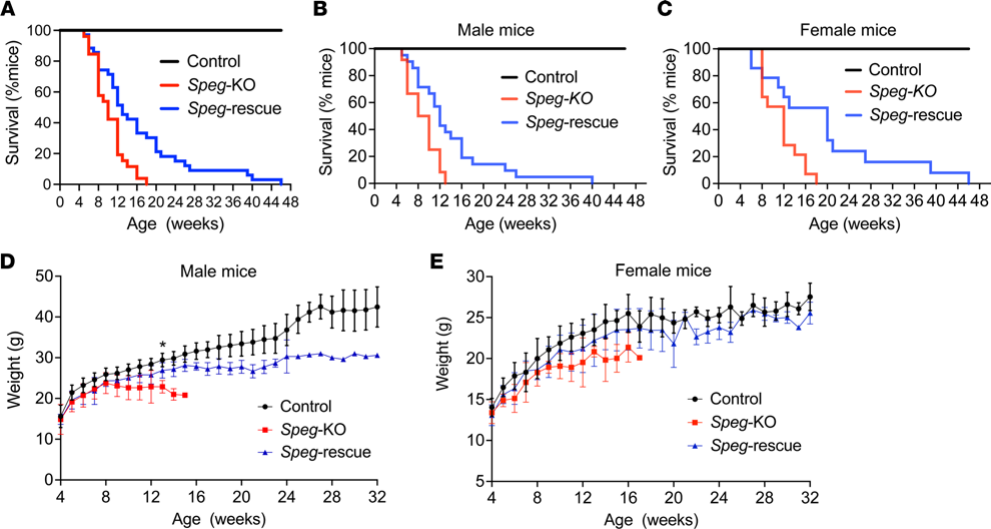
DNM2 reduction increases the life span and whole-body weight in *Speg*-rescue mice. (**A**) Life span of all *Speg*-rescue, *Speg*-KO, and control mice was monitored until 48 weeks of age. Data were represented as percentage survival (control, *n* = 35; *Speg*-KO, *n* = 26; *Speg*-rescue, *n* = 35). Survival rate for male (**B**) and female mice (**C**) were shown separately (for males: control, *n* = 21; *Speg*-KO, *n* = 12; *Speg*-rescue, *n* = 21; for females: control, *n* = 14; *Speg*-KO, *n* = 14; *Speg*-rescue, *n* = 14). Body weight of male (**D**) and female mice (**E**) was measured at different time points once per week and monitored until 32 weeks (for males: control, *n* = 14; *Speg*-KO, *n* = 6; *Speg*-rescue, *n* = 15; for females: control, *n* = 12; *Speg*-KO, *n* = 9; *Speg*-rescue, *n* = 10). The average body weight of male *Speg*-rescue mice begins to differentiate from that of *Speg*-KO mice at 13 weeks of age (*P ˂ 0.05; 1-way ANOVA with Tukey’s post hoc test) and remains higher afterward, while the average body weight of female *Speg*-KO and *Speg*-rescue mice is not significantly different at most of the time points.

The life span of *Speg*-rescue, *Speg*-KO, and control mice was monitored until 48 weeks of age, and their weight was checked once per week. We found that a reduction in DNM2 significantly improved the survival rate of *Speg*-KO mice (Figure 3A, *P* < 0.01). Although 100% of *Speg*-KO mice died by 18 weeks of life, 30% of *Speg*-rescue mice lived beyond that time (Figure 3A). The median ages of survival for male and female *Speg*-KO mice were 8 and 12 weeks, which increased to 12 and 20 weeks, respectively, in *Speg*-rescue mice (Figure 3, B and C). A higher body weight was also observed for *Speg*-rescue mice than that of KO mice, although body weight of male rescue mice was still significantly reduced compared with control mice. As an example, the average mouse weight at 13 weeks was 22.9 ± 1.6 g for male KO mice versus 26.9 ± 1.9 g for male rescue mice (*P* < 0.05) (Figure 3, D and E, and Supplemental Figure 4). These findings show that DNM2 reduction can partially improve the life span and growth delay observed in *Speg*-KO mice.

Interestingly, sex-related differences were observed in survival with a longer maximum life span seen in both female KO (~18 weeks) and female rescue (~46 weeks) mice compared with males (~13 weeks in *Speg*-KO and ~40 weeks in *Speg*-rescue mice). This sex-related difference may be related to hormonal influences and a higher expression of MCK in male mice (31–33).

### Improved motor function and increase in fiber size.

**Figure 4:**
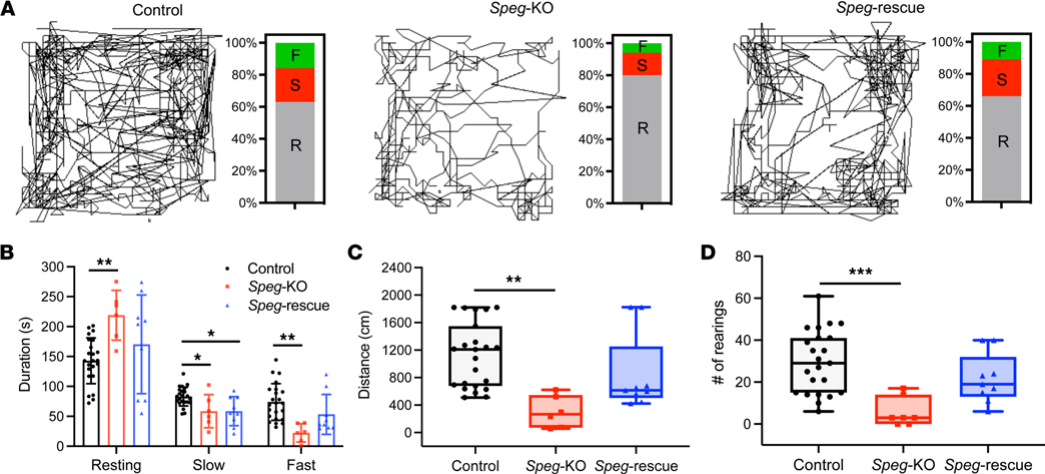
DNM2 reduction improves mouse motor function of *Speg*-KO mice. (**A**) Representative mouse activity maps from each genotype of mice and the corresponding activity type distribution graphs at 2 months of age. A clear improvement in ability to explore the arena is observed in *Speg*-rescue mice as compared with *Speg*-KO mice. R, resting time; S, slow movement; F, fast movement. (**B**) Distribution of mouse resting time, slow movement, and fast movement. Although not statistically significant, (**C**) *Speg*-rescue mice travel a longer horizontal distance (*P* = 0.08) than *Speg*-KO mice and (**D**) exhibit increased hind limb rearing behavior (*P* = 0.07) compared with *Speg*-KO mice. Control, *n* = 23; *Speg*-KO, *n* = 6; *Speg*-rescue, *n* = 9. *P < 0.05; **P < 0.01; ***P < 0.001; 1-way ANOVA with Tukey’s post hoc test.

To evaluate if DNM2 reduction improves overall activity, *Speg*-KO, *Speg*-rescue, and control mice were placed in a novel open-field activity box. Their motor function, including locomotor and rearing activities, were measured monthly at 2 and 3 months of age (Figure 4). Figure 4A shows the representative activity (fast, slow, and resting) of control, *Speg*-KO, and *Speg*-rescue mice. *Speg*-KO mice spent significantly more time resting and significantly less time moving fast compared with control mice (Figure 4B). Further, the *Speg*-rescue mice spent less time resting and more time moving fast compared with *Speg*-KO mice, although this did not reach statistical significance (Figure 4B). *Speg*-KO mice traveled a significantly shorter distance (Figure 4C) and reared significantly less frequently compared with control mice (Figure 4D). The travel distance and rearing frequency of *Speg*-rescue mice fell between the control and *Speg*-KO. *Speg*-rescue mice traveled a longer distance compared with *Speg*-KO mice (848 ± 561 vs. 302 ± 230, *P* = 0.08), albeit this difference was not statistically significant (Figure 4C). Additionally, *Speg*-rescue mice reared more frequently than *Speg*-KO mice (22 ± 12 vs. 6 ± 7, *P* = 0.07, Figure 4D). Thus, these data demonstrate that the mouse activity, distance, and number of rearings in *Speg*-rescue mice were improved compared with *Speg*-KO mice.

**Figure 5:**
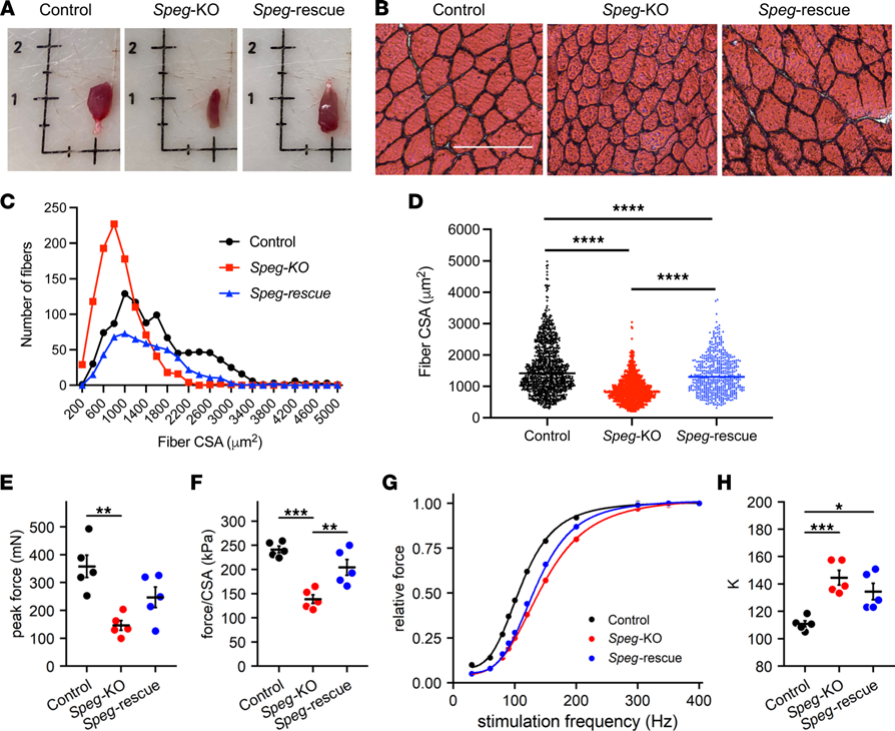
DNM2 reduction rescues skeletal muscle histology and improves contractility in extensor digitorum longus muscles of *Speg*-rescue mice. (**A**) Representative TA muscle images of control, *Speg*-KO, and *Speg*-rescue mice at 3 months of age. (**B**) H&E stains of TA muscles from control, *Speg*-KO, and *Speg*-rescue mice at 3 months of age. Scale bar: 100 μm. (**C**) Distribution of the cross-sectional area (CSA) in control, *Speg*-KO, and *Speg*-rescue TA muscles (*n* = 4 per group). (**D**) The mean CSA of *Speg*-rescue TA muscles is significantly larger than that of *Speg*-KO (****P < 0.0001, over 500 fibers were analyzed from each group; 1-way ANOVA with Tukey’s post hoc test). (**E**) Absolute peak tetanic force was significantly reduced in EDL muscles from *Speg*-KO mice compared with muscles from control animals. Muscles from the *Speg*-rescue mice were not different from either of the other groups. (**F**) Peak tetanic force expressed relative to EDL physiological CSA. Peak force/CSA was significantly reduced in *Speg*-KO animals (compared with control) but was restored to control levels in the *Speg*-rescue group. (**G**) Force-frequency relationships of EDL muscles. Forces obtained at different frequencies of stimulation were expressed relative to peak force and fit by the equation P_min_ + ([P_max_ – P_min_]/[1+([K/Hz]^H^)]), where P_min_ is the minimum force, P_max_ is the maximum force, K is the frequency corresponding to the inflection point of the curve, and H is a unitless parameter defining the curve’s slope. (**H**) The parameter K was significantly greater for the *Speg*-KO and the *Speg*-rescue EDL muscles compared with control, indicating a significant shift of the *Speg*-KO curve to the right. The parameter H did not differ between control (4.05 ± 0.21), *Speg*-KO (3.74 ± 0.04), and *Speg*-rescue (4.32 ± 0.22) muscles. *P ˂ 0.05; **P ˂ 0.01; ***P ˂ 0.001, *n* = 5 per genotype; 1-way ANOVA with Tukey’s post hoc test.

*Speg*-rescue had larger TA muscles compared with *Speg*-KO mice (Figure 5A). Further, while the fibers from *Speg*-KO TA were for the most part small and rounded (Figure 5B), the *Speg*-rescue TA fibers were larger and comparable in size and morphology to the control mice. Fiber cross-sectional area (CSA) distribution in *Speg*-KO mice was shifted toward smaller fibers (peak CSA, ~800 μm^2^), whereas it ranged from 1000 to 1500 μm^2^ in *Speg*-rescue mice, similar to that of control mice (Figure 5C). The mean fiber CSA in *Speg*-rescue mice was also significantly increased compared with *Speg*-KO mice (1384 ± 596 μm^2^ vs. 900 ± 405 μm^2^, *P* < 0.0001, Figure 5D).

### Restoration of contractile function of hind limb muscles.

To evaluate the contractility in skeletal muscle, the extensor digitorum longus (EDL) muscles from control, *Speg*-KO, and *Speg*-rescue mice were studied using an in vitro preparation. The absolute peak tetanic force of the EDL was significantly depressed (Figure 5E, *P* < 0.01) in *Speg*-KO mice. Force of *Speg*-rescue muscles fell between control and *Speg*-KO values, suggesting a partial restoration of contractile function. To further examine this potential therapeutic effect, force was normalized to CSA of each muscle (Figure 5F). Per unit CSA, EDL muscles of *Speg*-KO mice produced only 57.3% the force of EDL muscles from control mice. Importantly, muscles of *Speg*-rescue animals attained a mean peak force that was significantly elevated (*P* < 0.01) above the mean of the untreated *Speg*-KO mice. Because this beneficial effect is on a per unit CSA basis, it suggests that DNM2 treatment targeted intracellular mechanisms underlying the functional deficit of the *Speg*-KO mice.

We previously reported that the *Speg*-KO EDL muscles produced less force than control through measuring the force and stimulation frequency curves (7). Therefore, in this study, we measured force at stimulation frequencies ranging from 30 to 400 Hz (Figure 5G). For each individual muscle, forces were expressed relative to the muscle’s peak force. The relationship between relative force and stimulation frequency was described by a sigmoid function, relative force = P_min_ + ([P_max_ – P_min_]/[1+([K/Hz]^H^)]), where P_min_ is the minimum force, P_max_ is the maximum force, K is the stimulation frequency that corresponds to the inflection point of the relationship, and H is a unitless parameter describing the slope of the relationship. The slopes of the curves (H) were similar for all groups. However, the force-frequency relationship for EDL muscles of *Speg*-KO mice was shifted to the right as indicated by a significant increase (*P* < 0.001) in the parameter K (146 ± 6 Hz, mean ± SE) compared with the control mice (111 ± 3 Hz) in Figure 5H. This means that for a given stimulation frequency, EDL muscles from *Speg*-KO mice on average produced a lesser proportion of their peak force than muscles from control mice. The force-frequency curve for the *Speg*-rescue mice fell between the control and *Speg*-KO curves. Thus, DNM2 reduction resulted in a partial shift of the *Speg*-KO force-frequency relationship back toward that of control mice.

### Normalized localization of triadic proteins.

**Figure 6:**
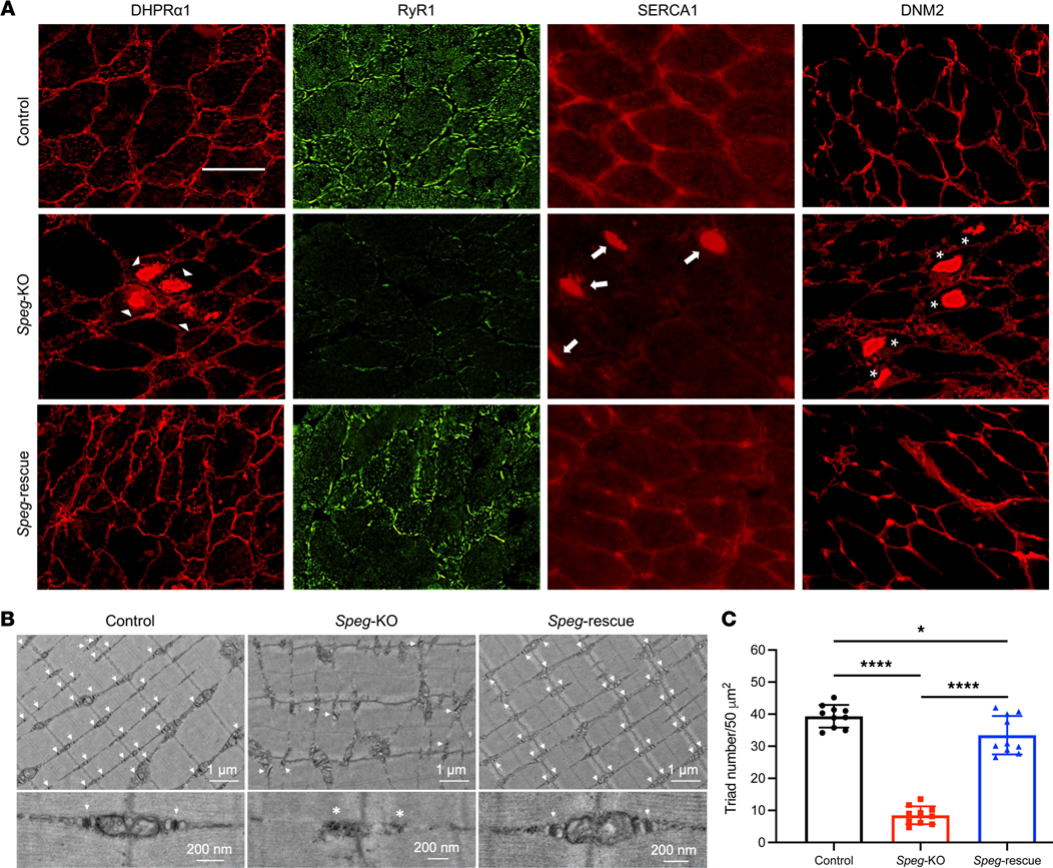
DNM2 reduction restores the localization of triadic proteins, triad ultrastructure, and triad number in *Speg*-rescue skeletal muscle. (**A**) Transverse TA muscle sections stained for DHPRα1, RyR1, SERCA1, and DNM2. Abnormally accumulated DHPRα1 (denoted by arrowheads), SERCA1 (denoted by arrows), and DNM2 (denoted by asterisks) were evident in discrete areas of *Speg*-KO myofibers yet absent in *Speg*-rescue mice (over 100 fibers were analyzed from each group, *n* = 3 per genotype). Scale bar: 50 μm. (**B**) Electron micrographs in quadriceps specimens obtained from control, *Speg*-KO, and *Speg*-rescue mice at 3 months of age. The upper panel shows an overall organization of muscle structure, and the lower panel shows an enlarged view of triad ultrastructure (white arrows) from each group. Abnormal and fewer triads (white asterisk) are noted in *Speg*-KO mice. (**C**) The number of triads per 50 μm^2^ was significantly decreased in *Speg*-KO mice compared with control mice. However, the triad number in *Speg*-rescue mice was remarkably increased compared with *Speg*-KO mice. Each dot represents a randomly selected field to count the triad number; *P ˂ 0.05; ****P < 0.0001; *n* = 3 per genotype; 1-way ANOVA with Tukey’s post hoc test.

We have previously shown that SPEG deficiency leads to abnormal distribution of multiple triadic proteins (34). To study the potential benefit of DNM2 reduction on the localization of triadic proteins, mouse TA muscles were stained with antibodies against DHPRα1 (marker for T-tubule), RyR1, SERCA1 (markers for terminal and longitudinal sarcoplasmic reticulum [SR]), and DNM2 (Figure 6A). We observed an abnormal accumulation of DHPRα1, SERCA1, and DNM2 in discrete areas of *Speg*-KO myofibers, indicating disorganized T-tubules and triads after SPEG depletion. However, these abnormalities observed in *Speg*-KO myofibers were not seen in *Speg*-rescue myofibers, suggesting that DNM2 reduction can normalize the abnormal localization of triadic proteins.

### Increase in triad number and improved triad ultrastructure.

To further evaluate the effects of DNM2 reduction on triad structure, sections of quadriceps muscle were examined using electron microscopy (EM). The *Speg*-KO muscle revealed structural triad abnormalities with regions of disoriented or absent triads, while *Speg*-rescue muscle displayed well-organized triad structure (Figure 6B). Additionally, quantitative analysis of 10 EM images revealed that triad density (the number of triads per 50 μm^2^) in *Speg*-rescue muscle was significantly improved (33.4 ± 6.0) compared with KO (8.5 ± 2.8) and approached control muscle (39.3 ± 3.5) (Figure 6C). Together, these findings suggest that DNM2 reduction improves the ultrastructure and number of triads in *Speg*-rescue skeletal muscle.

### SPEG deficiency leads to increase in phosphatidylinositol-3-phosphate levels and reduction in MTM1, whereas reducing DNM2 restores phosphatidylinositol-3-phosphate levels but not MTM1.

**Figure 7:**
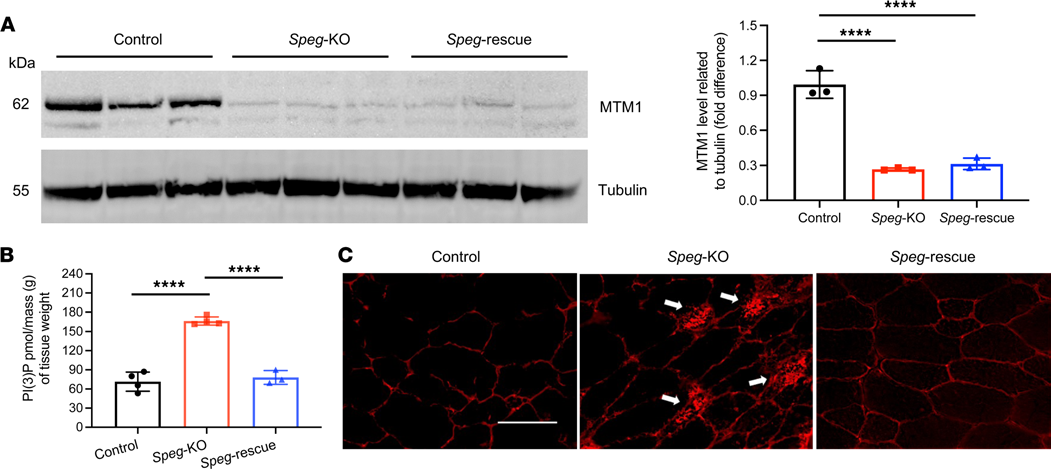
Restoration of PI3P levels in the *Speg*-rescue mice. (**A**) Immunoblot and quantification of MTM1 expression in triceps (****P ˂ 0.0001, *n* = 3 per genotype; 1-way ANOVA with Tukey’s post hoc test). (**B**) PI3P levels are increased in *Speg*-KO muscle, while PI3P levels in *Speg*-rescue muscle are similar to control muscle, as determined using a PI3P ELISA kit [purified lipid (pmol)/mass (g) of quadriceps muscle]. PI3P levels in control muscle = 71.5 ± 15.1 pmol/g (*n* = 4), *Speg*-KO muscle = 166.5 ± 6.5 pmol/g (*n* = 4), and *Speg*-rescue muscle = 78.1 ± 10.8 pmol/g (*n* = 3). ****P < 0.0001; 1-way ANOVA with Tukey’s post hoc test. (**C**) Immunostaining for PI3P on TA muscle. Abnormal PI3P accumulation (denoted by arrows) was detected in discrete areas of *Speg*-KO myofibers yet absent in *Speg*-rescue mice. Scale bar: 50 μm; over 100 fibers were analyzed from each group, *n* ≥ 3 per genotype.

SPEG interacts with MTM1 (5, 35), a lipid phosphatase that catalyzes the dephosphorylation of phosphatidylinositol-3-phosphate (PI3P) and phosphatidylinositol-3,5-bisphosphate (36, 37) and regulates membrane trafficking between endosomal and secretory compartments (38). We investigated the impact of SPEG deficiency on MTM1 expression in skeletal muscle using Western blot. The skeletal muscle samples obtained from both *Speg*-KO and *Speg*-rescue mice had significantly lower MTM1 protein levels (*P* < 0.0001) compared with control at 12 weeks (Figure 7A).

Loss of MTM1 results in an increased level of PI3P in human and animal skeletal muscles (39–42). We next measured PI3P levels in quadriceps extracted from control, *Speg*-KO, and *Speg*-rescue mice. The PI3P levels were similar in control and rescue mice, but they were significantly elevated (*P* < 0.0001) in *Speg*-KO mice (Figure 7B). To confirm this finding, we stained mouse TA muscles with PI3P antibody and observed accumulation of PI3P (Figure 7C) in *Speg*-KO myofibers, which normalized in *Speg*-rescue mice. Overall, this suggests that DNM2 reduction restores PI3P levels but not MTM1 in *Speg*-rescue mice.

### DNM2 reduction does not rescue the cardiac function.

Patients carrying recessive deleterious *SPEG* mutations present with CM, DCM, or both (3, 13). *Speg*-KO mice demonstrated cardiac dysfunction and evidence of increased left ventricular internal diameter and heart-to-body weight ratio (43). To evaluate the effects of DNM2 reduction on cardiac functions, we performed echocardiogram (echo) on *Speg*-KO, rescue, and control mice (Supplemental Figure 5A). The function of the left ventricle was assessed by measuring ejection fraction (Supplemental Figure 5B) and fractional shortening (Supplemental Figure 5C) at about 3 months of age. *Speg*-rescue mice showed no improvement in cardiac function compared to *Speg*-KO mice. Hearts were harvested after echo, and representative images of hearts from each group of mice were obtained (Supplemental Figure 5D). Notably, the heart of *Speg*-rescue mice was enlarged. We also measured the cardiac function of surviving *Speg*-rescue mice at 9 months of age and found that it was severely impaired (Supplemental Figure 6) compared with litter-matched control mice, indicative of DCM in older *Speg*-rescue mice.

To exclude the possibility that DNM2 reduction alone may affect the cardiac function in mice, 2 pairs of *Dnm2*^+/–^ and litter-matched control mice (1 male and 1 female from each group) at 5 months of age were selected for echo (Supplemental Figure 7A). We found that ejection fraction (Supplemental Figure 7B) and fractional shortening (Supplemental Figure 7C) were comparable in both groups of mice. Additionally, the heart size (Supplemental Figure 7D) and the heart-to-body weight ratio (mg/g) of *Dnm2*^+/–^ and litter-matched control mice were similar (*Dnm2*^+/–^ 7.7 ± 0.7 vs. control 6.0 ± 1.1). These findings suggest DNM2 reduction has no effect on cardiac function.

## Discussion

Recessive variants in *SPEG* cause a severe human disease with skeletal muscle (CNM) and cardiac (DCM) phenotypes. Developing precise therapies against *SPEG* mutations is complicated by SPEG’s large size, heterogeneity of the variants, and involvement of various tissues (skeletal muscle and heart). In this study, we evaluated DNM2 reduction as a potential strategy to rescue defects associated with SPEG deficiency. An investigational antisense oligonucleotide–based drug to reduce DNM2 levels and rescue phenotypes associated with *MTM1* and *BIN1* mutations is in phase I/II clinical trials (NCT04033159, https://clinicaltrials.gov, 2022) and could be easily adapted for the SPEG-related phenotype.

We have previously characterized a striated muscle-specific SPEG-deficient mouse model that recapitulates human disease (7, 43) with disruption of the triad structure and calcium homeostasis in skeletal muscles (7, 44). In this study, we demonstrate that SPEG-β interacted with DNM2, and loss of SPEG led to an increase in DNM2, similar to *Mtm1*- and *Bin1*-KO mouse models (16, 18). Reduction of DNM2 in *Speg*-KO mice was associated with an increased life span, improved body weight, amelioration of motor behavior, and alleviated myopathy-associated pathological features. We also observed a significant improvement in the force-frequency relationship (which is sensitive to impaired excitation-contraction coupling) and peak force per unit muscle CSA in *Speg*-rescue mice (Figure 5, F–H). Additionally, the localization of triadic proteins (DHPRα1, RyR1, and SERCA1), triad number, and triad ultrastructure were normalized in *Speg*-rescue mice. These findings suggest that reducing DNM2 may serve as a therapeutic strategy for SPEG-related myopathy.

Prior research elucidates a functional network in which MTM1, BIN1, and DNM2 interact with one another to regulate triad formation through a mechanism of membrane trafficking and remodeling (4, 16, 18, 25, 45). The interaction of BIN1 with DNM2 inhibits DNM2’s GTPase activity to promote membrane tubulation over fission during T-tubule development (18, 26), and MTM1 binding to BIN1 enhances tubulation activity (46). MTM1 also generates phosphatidylinositol-5-phosphate, a lipid precursor that is converted to phosphatidylinositol-4,5-bisphosphate by type II PI-5-P 4-kinases, and this conversion is critical for the recruitment of both BIN1 and DNM2 to the T-tubule membrane (36, 47). Mutations in *DNM2* and *MTM1* give rise to CNM phenotypes that greatly overlap with *SPEG*-related CNM (Table 1), including defects in triad formation and elevated levels or activity of DNM2.

We have previously demonstrated that SPEG interacts with the phosphatase and coiled-coil domains of MTM1 (5), and its deficiency causes defects in triad formation, similar to those seen in *Mtm1*-KO mice (40, 45, 48). In this study, we detected a marked reduction of MTM1 protein levels and an increased PI3P level in *Speg*-KO mice, which was also observed in *Mtm1*-KO mice (39, 40). These findings suggest that SPEG may play a role in stabilizing MTM1 protein, thereby regulating its phosphatase activity and/or function in the processes of endosomal trafficking, autophagy, and proteasome degradation. Additionally, we show that SPEG interacted with DNM2, and lowered DNM2 levels markedly improved triadic proteins’ localization, ultrastructure, and number and normalized the PI3P level in *Speg*-KO mice. The elevation of DNM2 level and the reduction of MTM1 associated with SPEG deficiency may disrupt a critical balance among endosomal dynamics, autophagy, and proteasomal degradation, which may be partially restored by DNM2 reduction (35, 49). These findings indicate shared molecular pathways in the regulation of triad development and maintenance, which deserve further investigation.

SPEG may have fiber type–specific and tissue-specific roles, which may affect the efficacy of DNM2 reduction in rescuing *Speg*-related myopathy and cardiomyopathy phenotypes. While DNM2 reduction could rescue the skeletal muscle defects and extend the life span in SPEG-deficient mice, it had little effect on improving cardiac function. A previous study reported that the protein expression of human SPEG-β was 65% greater in MyHC type IIx fibers than type I (50). We have previously found that the CSA and force of *Speg*-KO soleus muscle (oxidative/type I predominant) was considerably less affected than those of *Speg*-KO EDL (glycolytic/type II) muscle in mice (7), though SPEG-β did co-immunoprecipitate with DNM2 in the soleus (Supplemental Figure 1). A previous study found that RyR2 and JPH2 (junctional membrane proteins in the heart) could bind to only SPEG-β (longer isoform) and SPEG-α (shorter isoform), respectively (51). Additionally, the interacting partners of SPEG differ between skeletal and cardiac muscle (13). The Ig-like/fibronectin type III domains of SPEG interact with MTM1 and desmin in skeletal muscle (5, 34), while the kinase domain of SPEG interacts with JPH2, RyR2, SERCA2a, and tropomyosins in cardiac muscle (6, 51–53). These findings suggest differential tissue-specific roles of SPEG isoforms. Indeed, patients with recessive mutations affecting both SPEG-α and SPEG-β demonstrate more severe clinical and molecular phenotypes, while patients with mutations affecting only SPEG-β are associated with a milder phenotype, and without cardiac involvement (13). The differential rescue response of DNM2 reduction in skeletal and cardiac muscles needs further exploration, and novel approaches are needed to overcome the cardiac dysfunction associated with SPEG deficiency.

In summary, we show that SPEG-β interacted with DNM2 in the skeletal muscle and that SPEG deficiency caused an increase in DNM2 levels. Reducing DNM2 could increase the life span, body weight, and motor performance of SPEG-deficient mice, thereby rescuing SPEG-related myopathy, but not alleviate the cardiac dysfunction. This suggests differential interaction among SPEG, MTM1, and DNM2 in skeletal and cardiac muscles. Combination therapeutic strategies should be considered to target both the cardiac and skeletal defects associated with SPEG deficiency. Further, DNM2 reduction should be explored as a therapeutic strategy against other genetic CMs (and dystrophies) that are associated with a higher level of DNM2.

## Methods

Additional methods are in Supplemental Methods. Additional data supporting the figures are in Supplemental Data 1.

### Study design.

*Speg*-KO mice were generated as previously described (7). Homozygous *Speg*–conditional KO mice (*Speg^ﬂ/ﬂ^*) were bred with male transgenic mice that have the Cre recombinase driven by MCK promoter (MCK-Cre^+^), with Cre activity observed in skeletal and cardiac muscle. Speg–conditional KO mouse generation was performed by inGenious Targeting Laboratory (Ronkonkoma, New York, USA), and MCK-Cre^+^ mice [B6.FVB(129S4)-Tg(Ckmm-cre)5Khn/J; strain 006475 were from The Jackson Laboratory (Bar Harbor, Maine, USA). *Dnm2*-heterozygous (*Dnm2^+/–^*) mice (16) were bred to generate the *Speg*-rescue (*Speg^fl/fl^ MCK-Cre^+^ Dnm2^+/–^*), *Speg*-KO (*Speg^fl/fl^ MCK-Cre^+^ Dnm2^+/+^*), and control (*Speg^fl/+^ MCK-Cre^+^ Dnm2^+/+^*, *Speg^fl/fl^ MCK-Cre^–^ Dnm2^+/+^*, or *Speg^fl/+^ MCK-Cre^–^ Dnm2^+/+^*) mice. Specific primers were used to identify *Speg^ﬂ/ﬂ^*, MCK-Cre^+^, and *Dnm2^+/–^* alleles (7, 16). The sample size for each experiment is included in the figure legends.

### Immunoblot analysis.

Skeletal muscles from control, *Speg*-KO, and *Speg*-rescue littermate mice were dissected, snap-frozen in isopentane, and stored at −80°C until analysis. Protein isolation and Western blot procedures were performed as described previously (54). Immunofluorescence Western blot was performed in addition to chemiluminescence Western blot. Proteins were probed with antibody against rabbit anti-SPEG (catalog 12472-T16, 1:1000 dilution, SinoBiological), mouse anti-DNM2 (catalog sc-166526, 1:100 dilution, Santa Cruz Biotechnology), rabbit anti-MTM1 (clone PI168, 1:700 dilution, from IGBMC), and mouse anti-GAPDH (catalog MA5-15738, 1:1000 dilution, Thermo Fisher Scientific). Secondary horseradish peroxidase–conjugated antibodies against rabbit (catalog 7074S, 1:2000 dilution, Cell Signaling Technology) and against mouse (catalog 7076S, 1:2000 dilution, Cell Signaling Technology) were detected using enhanced chemiluminescence. IRDye 800CW donkey anti-rabbit IgG secondary antibody (catalog 926-32213, 1:5000, LI-COR), IRDye 680RD donkey anti-mouse IgG secondary antibody (catalog 926-68072, 1:5000, LI-COR), anti-tubulin rhodamine antibody (clone AbD22584, 1:5000, Bio-Rad Laboratories), and anti-GAPDH rhodamine antibody (catalog 12004168, 1:5000, Bio-Rad Laboratories) were used for immunofluorescence detection. Quantification of protein levels normalized to GAPDH or tubulin was performed using ImageJ software (NIH).

### co-IP.

Lysates from C2C12 myotubes, soleus, and triceps were obtained by homogenization in co-IP buffer (10% NP-40, 20% 20 mM NaF, 1% Triton X-100) supplemented with complete protease inhibitor tablet (Roche Applied Science) and 1 mM leupeptin and 1 mM pepstatin A (MilliporeSigma). Cells were collected and lysed at 4°C for 30 minutes. After centrifugation (16,000*g*, 4°C, 20 minutes), the soluble fractions were collected, and the concentration was measured using a colorimetric BCA assay (23225; Thermo Fisher Scientific). Soluble homogenates were precleared with Dynabead Protein G beads (Thermo Fisher Scientific) for 1 hour, and supernatants were incubated with the specific antibodies directed against the protein of interest at 4°C for 12 to 24 hours. Dynabead Protein G beads were then added for 2 hours to capture the immune complex. Beads were washed 3 times with co-IP buffer supplemented with 0.1% CHAPS. For all experiments, 2 negative controls consisted of a sample lacking the primary antibody and a sample incubated with another primary antibody from the same serotype as the antibody of interest. Resulting beads were eluted with Laemmli buffer and subjected to SDS-PAGE followed by immunoblot.

### Behavioral testing.

Locomotor and behavioral activity were evaluated using ActiTrack tracking software (V2.7, Panlab, S.L.U.) as previously described (7). Analysis of breaks in infrared light beams was used by the software to record activity, position, rearings, and speed. Mice were allowed to freely explore the arena over a 5-minute period, and distance traveled, speed of movement, and number of rearings were used to evaluate muscle function. Testing was done in the afternoon at approximately the same time for each session. Default movement speed thresholds were used to evaluate movement speed, where movement of less than 2 cm/s was considered resting, movement between 2 and 5 cm/s was considered slow speed, and movement of more than 5 cm/s was considered fast speed.

### Histology and immunofluorescence.

Cross sections (8 μm thick) of isopentane-frozen TA were stained with H&E using standard techniques. M.O.M. (Mouse on Mouse) Blocking Reagent (MKB-2213-1, Vector Laboratories) was used to block the endogenous mouse Ig staining. Samples were stained with goat anti-mouse IgG (H+L) secondary antibody–Alexa Fluor 594 (catalog A-11005, 1:1000 dilution, Thermo Fisher Scientific) and goat anti-rabbit IgG (H+L) secondary antibody–Alexa Fluor 488 (catalog A-11008, 1:1000 dilution, Thermo Fisher Scientific) to exclude the nonspecific staining. Immunofluorescence was performed by standard protocol using mouse anti-DHPRα1 antibody (CACNA1S, catalog ab2862, 1:50 dilution, Abcam), rabbit anti-RyR1 (1:100 dilution, from Isabelle Marty, University Grenoble Alpes, INSERM, Grenoble, France), mouse anti-SERCA1 (catalog ab2819, 1:500 dilution, Abcam), mouse anti-DNM2 (catalog sc-166526, 1:50 dilution, Santa Cruz Biotechnology), and mouse anti-PI3P (catalog Z-P003, 1:50 dilution, Echelon Biosciences) for primary antibodies. Goat anti-mouse IgG (H+L) secondary antibody–Alexa Fluor 594 (catalog A-11005, 1:1000 dilution, Thermo Fisher Scientific) and goat anti-rabbit IgG (H+L) secondary antibody–Alexa Fluor 488 (catalog A-11008, 1:1000 dilution, Thermo Fisher Scientific) were used for secondary antibodies. Slides were coverslipped using EverBrite mounting medium (23001, Biotium). Images were captured using a Nikon Eclipse 90i microscope in conjunction with NIS-Elements AR software (Nikon Instruments Inc.).

### Muscle contractility.

The functional properties of skeletal muscles of control, *Speg*-KO, and *Speg*-rescue mice were directly assessed using the ex vivo methods described in our earlier work with this model (7). EDL muscles were dissected and attached via their tendons to the lever arm of a dual-mode muscle lever system (Aurora Scientific, model 300B-LR) and a stationary post. The muscles were submerged in a temperature-controlled (35°C) bicarbonate buffer continuously equilibrated with 95% O_2_, 5% CO_2_.

Contractions were induced via output from a biphasic muscle stimulator delivered to platinum electrodes flanking the preparation. Data were collected at the muscle length (optimal length) that maximized tetanic force (300 Hz). Muscles were stimulated with trains of square wave pulses of increasing frequency (from 10 up to 400 Hz). The force-frequency data were fit by a sigmoidal curve as described previously (7), yielding parameters specifying the minimal force (equivalent to twitch force), maximum force (equivalent to peak tetanic force), inflection point of the curve, and curve’s slope. Physiological CSA area, used to normalize tetanic force, was calculated as mass/(fiber length × muscle density). Fiber length was calculated as muscle optimal length × 0.44, where 0.44 is the fiber length–to–muscle length ratio of the EDL (55). Muscle density was taken as 1.06 mg/mm^3^ (56).

### Transmission EM.

Skeletal muscle samples of quadriceps (1–2 mm cubes) were fixed in 2.5% glutaraldehyde, 1.25% paraformaldehyde, and 0.03% picric acid in 0.1 M sodium cacodylate buffer (pH 7.4) overnight at room temperature and stored at 4°C. They were then washed in 0.1 M cacodylate buffer and postfixed with 1% osmium tetroxide/1.5% potassium ferrocyanide for 1 hour, washed in water 3 times, and incubated in 1% aqueous uranyl acetate for 1 hour followed by 2 washes in water and subsequent dehydration in grades of alcohol (10 minutes each; 50%, 70%, 90%, twice for 10 minutes 100%). The samples were then put in propylene oxide for 1 hour and infiltrated overnight in a 1:1 mixture of propylene oxide and TAAB Epon (Marivac Canada Inc.). The following day, the samples were embedded in TAAB Epon and polymerized at 60°C for 48 hours. Ultrathin sections (~60 nm) were cut on a Reichert Ultracut-S microtome, picked up onto copper grids stained with lead citrate, and examined in a JEOL 1200EX Transmission electron microscope, and images were recorded with an AMT 2k charge-coupled device camera. This was performed at the Electron Microscopy Facility of Harvard Medical School.

### PI3P ELISA.

PI3P Mass ELISA was performed on lipid extracts from whole quadriceps skeletal muscle preparations according to the manufacturer’s recommendations (K-3300, Echelon Biosciences). Briefly, quadriceps were isolated, weighed, ground in a mortar and pestle under liquid nitrogen, and homogenized before the addition of ice-cold 0.5 M trichloroacetic acid to extract lipids. Extracted lipids were transferred into a clean Eppendorf tube and dried in a vacuum dryer. PI3P extraction samples were resuspended in PBS-Tween with 3% protein stabilizer and then spotted on PI3P Mass ELISA plates in triplicate. PI3P levels were detected by measuring absorbance at 450 nm on a plate reader according to the protocol.

### Statistics.

Results were analyzed with GraphPad Prism (v.8.0; GraphPad Software) and expressed as mean ± SD. Survival curve was analyzed with GraphPad Prism using the log rank test (Mantel-Cox test). Unpaired 2-tailed *t* test was used to determine statistically significant differences for 2-group comparisons. One-way ANOVA followed by Tukey’s post hoc test was used for multiple-group comparisons. The numbers of samples per group (*n*) and statistical significance for all comparisons are specified in the figure legends. *P* < 0.05 was considered statistically significant.

### Study approval.

All studies were approved by the Institutional Animal Care and Use Committee at Children’s Hospital Boston (approval number 20-05-4179). The work followed the *Guide for the Care and Use of Laboratory Animals* (National Academies Press, 2011) and all the regulatory protocols set forth by the Boston Children’s Hospital Animal Resources at Children’s Hospital facility.

## Author contributions

QL and PBA designed the experiments and performed project administration. QL, J Lin, JJW, SL, GL, YZ, J Laporte, MAP, XL, and PBA carried out experiments, performed data analyses, and drafted the manuscript. All authors read and approved the final manuscript.

## Supplementary Material

Supplemental data

Supplemental data set 1

## Figures and Tables

**Table 1:**
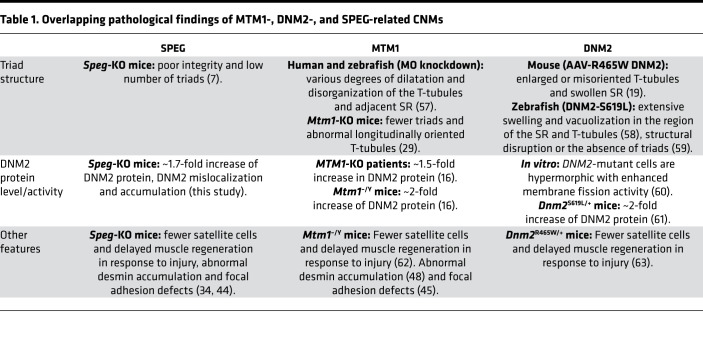
Overlapping pathological findings of MTM1-, DNM2-, and SPEG-related CNMs

## Version 1 06/28/2022

In-Press Preview

## Version 2 08/08/2022

Electronic publication
