# Supplementary material for: Dynamin-2 reduction rescues the skeletal myopathy of a SPEG-deficient mouse model
Source: JCI Insight. 2022 Aug 8;7(15):e157336. doi: 10.1172/jci.insight.157336 (PMC9462472; doi:10.1172/jci.insight.157336)
Supplement: Supplemental data [file jciinsight-7-157336-s018.pdf]

Dynamin-2 reduction reduces the skeletal myopathy of SEPG-deficient mouse model

Qifei Li[1,2,3], Jasmine Lin[1,2,3], Jeffrey J. Widrick[2,3], Shiyu Luo[1,2,3], Gu Li[4,5], Yuanfan Zhang[2], Jocelyn Laporte[6], Mark A. Perrella[4,5], Xiaoli Liu[4,5], Pankaj B. Agrawal[1,2,3,*]

[1]Division of Newborn Medicine, [2]Division of Genetics and Genomics, [3]The Manton Center for Orphan Disease Research, Boston Children's Hospital, Harvard Medical School; Boston, Massachusetts 02115, USA.

[4]Division of Pulmonary and Critical Care Medicine, [5]Department of Pediatric Newborn Medicine, Brigham and Women's Hospital, Harvard Medical School; Boston, Massachusetts 02115, USA.

[6]IGBMC (Institut de Génétique et de Biologie Moléculaire et Cellulaire), INSERM U1258, CNRS UMR7104, Strasbourg University, 67404 Illkirch, France.

*To whom correspondence should be addressed at: Pankaj B. Agrawal, MD, Boston Children's Hospital and Harvard Medical School, 300 Longwood Ave., Enders 9, Boston, MA 02115, USA. Tel: +1 6179192153; Fax: +1 6177300486; Email: pagrawal@enders.tch.harvard.edu

Supplementary Materials

Methods

Immunoblot Analysis. Skeletal muscles from control, *Speg*-KO and *Speg*-rescue littermate mice were dissected, snap frozen in isopentane, and stored at −80°C until analysis. Protein isolation and western blot procedures were performed as described previously(1). Immunofluorescent western blot was performed in addition to chemiluminescent western blot. Proteins were probed with antibody against rabbit anti-SPEG (12472-T16, 1:1000 dilution, SinoBiological, Beijing, China), mouse anti-DNM2 (sc-166526, 1:100 dilution, Santa Cruz Biotechnology), and mouse anti-glyceraldehyde-3-phosphate dehydrogenase (GAPDH; MA5-15738, 1:1000 dilution, ThermoFisher Scientific). Secondary horseradish peroxidase-conjugated antibodies against rabbit (7074S, 1:2000 dilution, Cell Signaling Technology) and against mouse (7076S, 1:2000 dilution, Cell Signaling Technology, Danvers, MA, USA) were detected using enhanced chemiluminescence. IRDye 800CW Donkey anti-Rabbit IgG Secondary antibody (926-32213, 1:5000, LI-COR), IRDye 680RD Donkey anti-Mouse IgG Secondary antibody (926-68072, 1:5000, LI-COR), and anti-GAPDH Rhodamine antibody (12004168, 1:5000, Bio-Rad Laboratories) were used for immunofluorescence detection. Quantification of protein levels normalized to GAPDH was performed using ImageJ software.

Echocardiography. Transthoracic echocardiography was performed on mice under isoflurane anesthesia, using a Vevo 2100 high-resolution micro ultrasound system and a 50 MHz probe. The hearts were imaged in the B-model for the two-dimensional

parasternal short-axis view at the level of papillary muscles (2, 3), and the M-mode for the end-systolic and end-diastolic internal dimensions of the LV (LVID, s; LVID, d, respectively). The LV fraction shortening (LVFS) and ejection fraction (LVEF) were calculated by the following formula, SF=(LVID,d-LVID,s)/LVID,d × 100, EF% = (LV vol, d-LV vol,s)/LV vol, d × 100. The echocardiograms were performed in a blinded manner, without knowing the genotype in advance.

Supplementary figures

Supplementary Figure 1. SPEGβ interacts DNM2 in skeletal muscle. SPEGβ and DNM2 coimmunoprecipitated from soleus and triceps lysates with the use of rabbit anti-SPEG generated against a FLAG-tagged APEG-1 fusion protein and anti-DNM2 antibodies.

Supplementary Figure 2. Breeding strategy of *Speg*-KO mice with DNM2 haploinsufficiency (*Speg*-rescue). *Speg*-KO mice ($Speg^{fl/fl}/MCK\text{-}Cre^{+}/Dnm2^{+/+}$) are cre-positive, homozygous for floxed *Speg* allele and DNM2 WT; *Speg*-rescue mice ($Speg^{fl/fl}/MCK\text{-}Cre^{+}/Dnm2^{+/-}$) are MCK-cre-positive, homozygous for floxed *Speg* allele and heterozygous for DNM2 allele; Control mice ($Speg^{fl/+}/MCK\text{-}Cre^{+}/Dnm2^{+/+}$, $Speg^{fl/fl}/MCK\text{-}Cre^{-}/Dnm2^{+/+}$ or $Speg^{fl/+}/MCK\text{-}Cre^{-}/Dnm2^{+/+}$) are DNM2 WT.

Supplementary Figure 3. DNM2 protein expression in striated muscles. Immunoblot images of SPEG and DNM2 expression in various types of striated muscles, including gastrocnemius, triceps, diaphragm, and heart.

Supplementary Figure 4. DNM2 reduction increases body weight of *Speg*-KO mice. (A) Comparison of body weight at 11 weeks for male mice (control, n = 14; *Speg*-KO, n = 4; *Speg*-rescue, n = 11). (B) Comparison of body weight at 15 weeks for female mice (control, n = 9; *Speg*-KO, n = 4; *Speg*-rescue, n = 5). *, $P < 0.05$; ***, $P < 0.001$; one-way ANOVA with Tukey's post hoc test.

Supplementary Figure 5. DNM2 reduction fails to rescue cardiac phenotype in *Speg*-KO mice. (A) Representative mouse echocardiograms at 3 months of age. The left ventricles of hearts were assessed for (B) ejection fraction (LVEF, %) and (C) fractional

shortening (LVFS, %) at 3 months of age (n = 3 per genotype; one-way ANOVA with Tukey's post hoc test). (D) Representative macroscopic images of hearts from each group of mice.

Supplementary Figure 6. ***Speg*****-rescue mice develop an enlarged heart with impaired cardiac function over time**. The left ventricles of hearts were evaluated for ejection fraction (LVEF, %) and fractional shortening (LVFS, %) at 9 months of age (n = 1 per genotype).

Supplementary Figure 7. **DNM2 haploinsufficient mice are absent of cardiac phenotype.** (A) Representative mouse echocardiograms of DNM2 haploinsufficient and litter-matched control mice at 5 months of age. The left ventricles of hearts were assessed for (B) ejection fraction (LVEF, %) and (C) fractional shortening (LVFS, %). (D) Images of hearts from DNM2 haploinsufficient and litter-matched control mice; n = 2 per genotype.

Supplementary Table 1. Breeding strategy and outcome for *Speg*-rescue mice (*$Speg^{fl/fl}$/MCK-Cre^{+}/$Dnm2^{+/-}$*) with expected mice and obtained at 21 days after birth.

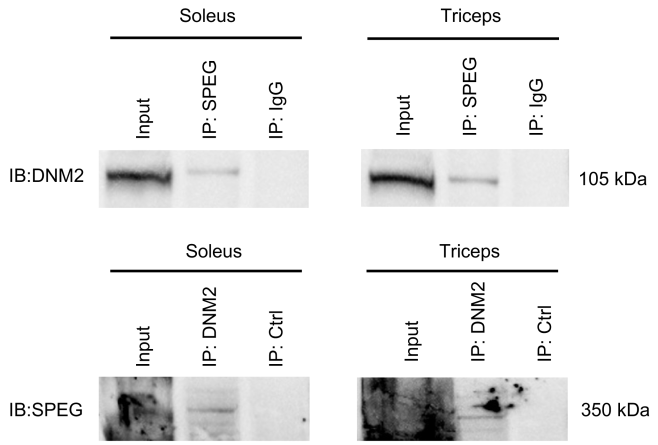

Supplementary Figure 1. SPEGβ interacts DNM2 in skeletal muscle. SPEGβ and DNM2 coimmunoprecipitated from soleus and triceps lysates with the use of rabbit anti-SPEG generated against a FLAG-tagged APEG-1 fusion protein and anti-DNM2 antibodies.

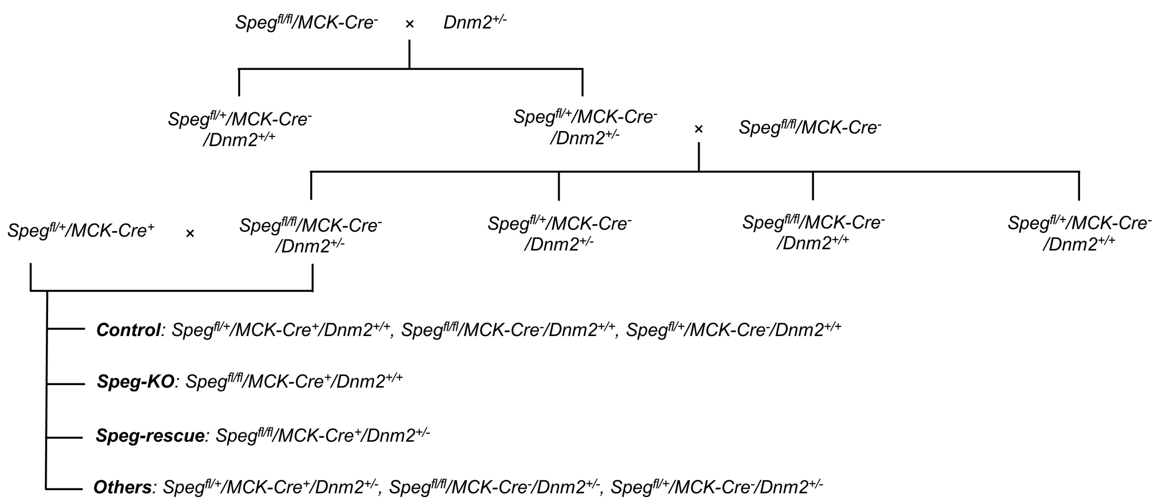

Supplementary Figure 2. **Breeding strategy of *Speg*-KO mice with DNM2 haploinsufficiency (*Speg*-rescue)**. *Speg*-KO mice ($Speg^{fl/fl}/MCK\text{-}Cre^{+}/Dnm2^{+/+}$) are cre-positive, homozygous for floxed *Speg* allele and DNM2 WT; *Speg*-rescue mice ($Speg^{fl/fl}/MCK\text{-}Cre^{+}/Dnm2^{+/-}$) are MCK-cre-positive, homozygous for floxed *Speg* allele and heterozygous for DNM2 allele; Control mice ($Speg^{fl/+}/MCK\text{-}Cre^{+}/Dnm2^{+/+}$, $Speg^{fl/fl}/MCK\text{-}Cre^{-}/Dnm2^{+/+}$ or $Speg^{fl/+}/MCK\text{-}Cre^{-}/Dnm2^{+/+}$) are DNM2 WT.

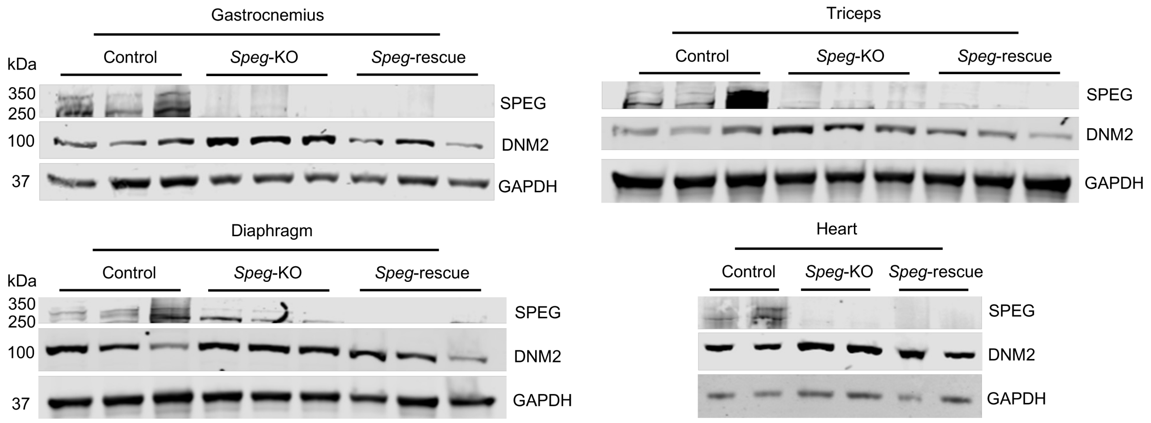

Supplementary Figure 3. **DNM2 protein expression in striated muscles**. Immunoblot images of SPEG and DNM2 expression in various types of striated muscles, including gastrocnemius, triceps, diaphragm, and heart.

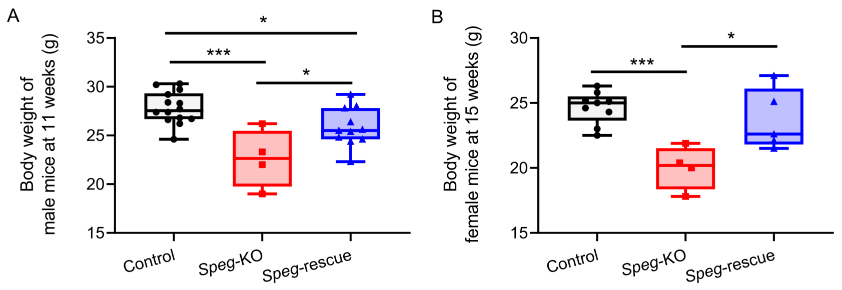

Supplementary Figure 4. **DNM2 reduction increases body weight of *Speg*-KO mice**. (A) Comparison of body weight at 11 weeks for male mice (control, n = 14; *Speg*-KO, n = 4; *Speg*-rescue, n = 11). (B) Comparison of body weight at 15 weeks for female mice (control, n = 9; *Speg*-KO, n = 4; *Speg*-rescue, n = 5). *, $P < 0.05$; ***, $P < 0.001$; one-way ANOVA with Tukey's post hoc test.

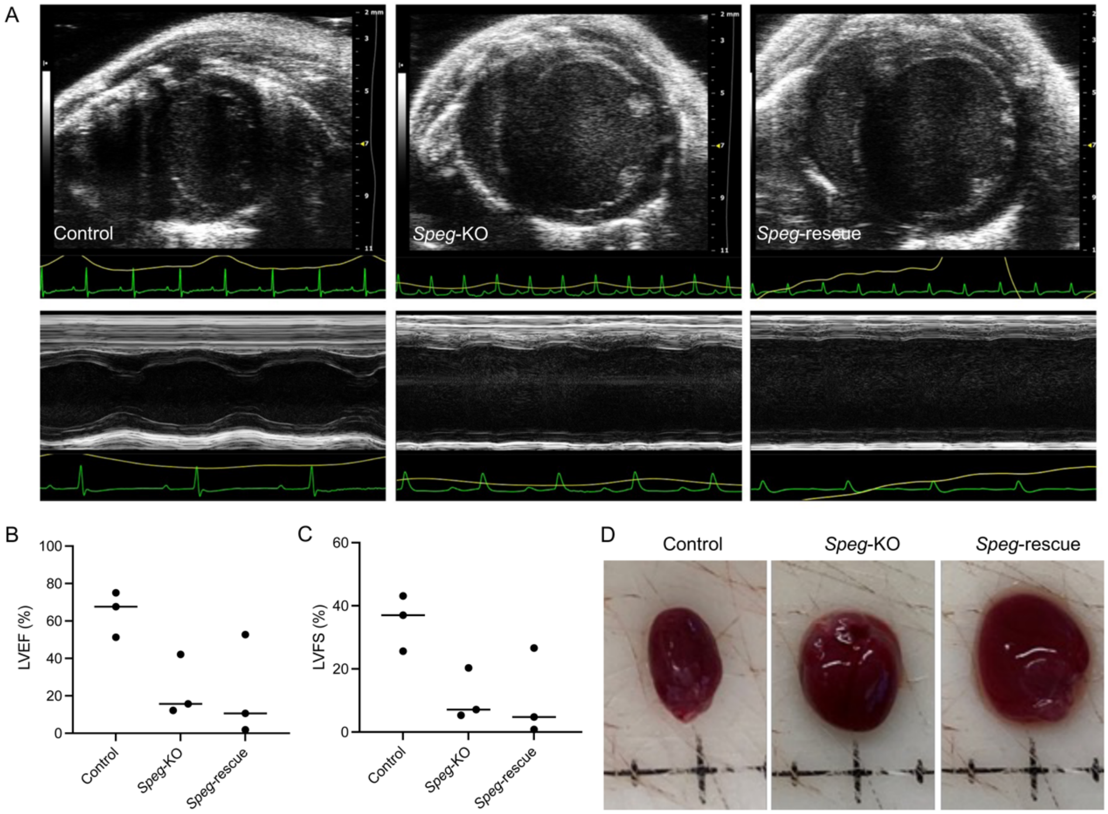

Supplementary Figure 5. **DNM2 reduction fails to rescue cardiac phenotype in *Speg*-KO mice.** (A) Representative mouse echocardiograms at 3 months of age. The left ventricles of hearts were assessed for (B) ejection fraction (LVEF, %) and (C) fractional shortening (LVFS, %) at 3 months of age (n = 3 per genotype; one-way ANOVA with Tukey's post hoc test). (D) Representative macroscopic images of hearts from each group of mice.

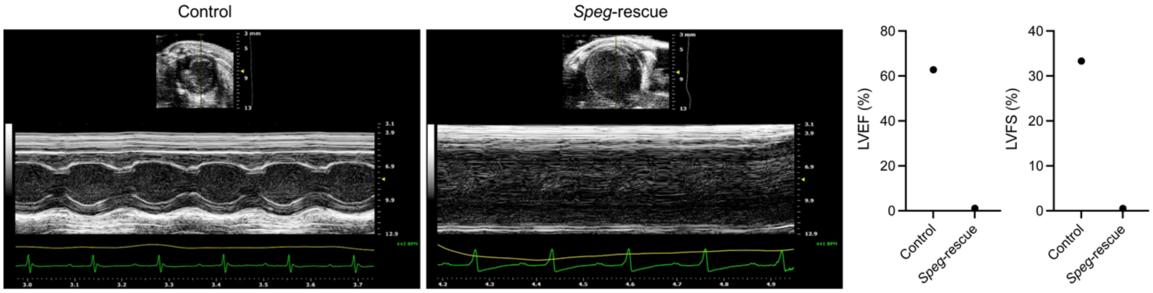

Supplementary Figure 6. ***Speg*-rescue mice develop an enlarged heart with impaired cardiac function over time**. The left ventricles of hearts were evaluated for ejection fraction (LVEF, %) and fractional shortening (LVFS, %) at 9 months of age (n = 1 per genotype).

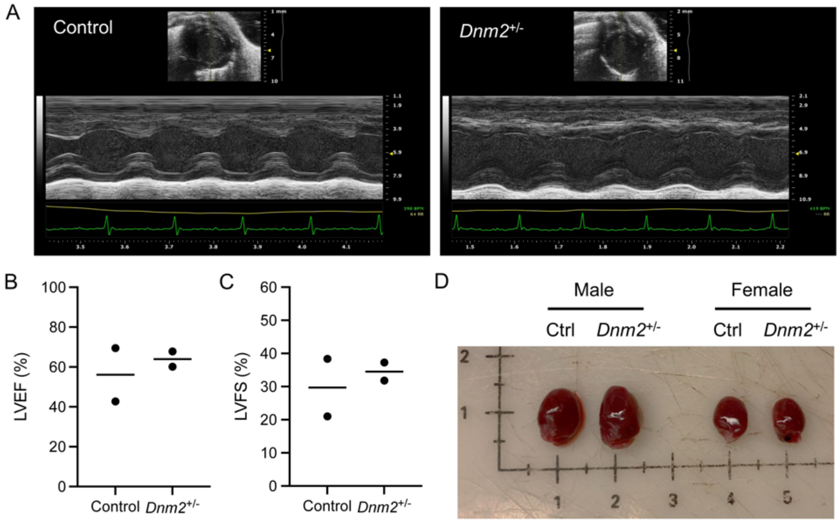

Supplementary Figure 7. **DNM2 haploinsufficient mice are absent of cardiac phenotype.** (A) Representative mouse echocardiograms of DNM2 haploinsufficient and litter-matched control mice at 5 months of age. The left ventricles of hearts were assessed for (B) ejection fraction (LVEF, %) and (C) fractional shortening (LVFS, %); n = 2 per genotype. (D) Images of hearts from DNM2 haploinsufficient and litter-matched control mice.

Supplementary Table 1. Breeding strategy and outcome for *Speg*-rescue mice (*Speg$^{fl/fl}$/MCK-Cre^{+}/Dnm2$^{+/-}$*) with expected mice and obtained at 21 days after birth.

Speg$^{fl/f}$/Dnm2$^{+/-}$ x *Speg$^{fl/+}$/MCK-Cre^{+}*				
Offspring (n =315)	*Speg$^{fl/fl}$/MCK-Cre^{+}/Dnm2$^{+/-}$* (*Speg*-rescue)	*Speg$^{fl/fl}$/MCK-Cre^{+}/Dnm2$^{+/+}$* *(Speg*-KO)	*Speg$^{fl/fl}$/MCK-Cre^{-}/Dnm2$^{+/+}$*, *Speg$^{fl/+}$/MCK-Cre^{-}/Dnm2$^{+/+}$*, *Speg$^{fl/+}$/MCK-Cre^{+}/Dnm2$^{+/+}$* (Control)	Others
Obtained genotypes	41	38	142	94
Expected genotypes	39.375	39.375	118.12	118.12

Others: *Speg$^{fl/fl}$/MCK-Cre^{-}/Dnm2$^{+/-}$*, *Speg$^{fl/+}$/MCK-Cre^{-}/Dnm2$^{+/-}$*, *Speg$^{fl/+}$/MCK-Cre^{+}/Dnm2$^{+/-}$*.

References

1. Agrawal PB, Joshi M, Savic T, Chen Z, and Beggs AH. Normal myofibrillar development followed by progressive sarcomeric disruption with actin accumulations in a mouse Cfl2 knockout demonstrates requirement of cofilin-2 for muscle maintenance. *Hum Mol Genet.* 2012;21(10):2341-56.
2. Liu X, Ramjiganesh T, Chen Y-H, Chung SW, Hall SR, Schissel SL, et al. Disruption of striated preferentially expressed gene locus leads to dilated cardiomyopathy in mice. *Circulation.* 2009;119(2):261-8.
3. Liao R, Jain M, Cui L, D'Agostino J, Aiello F, Luptak I, et al. Cardiac-specific overexpression of GLUT1 prevents the development of heart failure attributable to pressure overload in mice. *Circulation.* 2002;106(16):2125-31.
